# Meeting the WHO 90% target: antiretroviral treatment efficacy in Poland is associated with baseline clinical patient characteristics

**DOI:** 10.7448/IAS.20.1.21847

**Published:** 2017-07-17

**Authors:** Milosz Parczewski, Ewa Siwak, Magdalena Leszczyszyn-Pynka, Iwona Cielniak, Ewa Burkacka, Piotr Pulik, Adam Witor, Karolina Muller, Ewelina Zasik, Anna Grzeszczuk, Maria Jankowska, Małgorzata Lemańska, Anita Olczak, Edyta Grąbczewska, Aleksandra Szymczak, Jacek Gąsiorowski, Bartosz Szetela, Monika Bociąga-Jasik, Paweł Skwara, Magdalena Witak-Jędra, Elżbieta Jabłonowska, Kamila Wójcik-Cichy, Juliusz Kamerys, Małgorzata Janczarek, Dagny Krankowska, Tomasz Mikuła, Katarzyna Kozieł, Dariusz Bielec, Justyna Stempkowska, Aleksandra Kocbach, Wiesława Błudzin, Andrzej Horban

**Affiliations:** ^a^ Pomeranian Medical University, Department of Infectious, Tropical Diseases and Immune Deficiency, Szczecin, Poland; ^b^ Hospital for Infectious Diseases, HIV Out-Patient’s Clinic, Warsaw, Poland; ^c^ Regional Hospital, Out-Patient’s Clinic for Immune Deficiency, Chorzów, Poland; ^d^ Department of Infectious Diseases and Hepatology, Medical University of Białystok, Białystok, Poland; ^e^ Department of Infectious Diseases, Medical University of Gdańsk, Gdańsk, Poland; ^f^ Department of Infectious Diseases and Hepatology Nicolaus Copernicus University Ludwik Rydygier Collegium Medicum in Bydgoszcz, Faculty of Medicine, Bydgoszcz, Poland; ^g^ Department of Infectious Diseases, Liver Disease and Acquired Immune Deficiencies, Wroclaw Medical University, Wrocław, Poland; ^h^ Department of Infectious Diseases, Jagiellonian University Medical College, Kraków, Poland; ^i^ Department of Infectious Diseases and Hepatology, Medical University of Łódź, Łódź, Poland; ^j^ Department of Infectious and Tropical Diseases and Hepatology, Medical University in Warsaw, Warsaw, Poland; ^k^ Department of Infectious Diseases, Regional Hospital in Zielona Góra, Zielona Góra, Poland; ^l^ Department of Infectious Diseases, Medical University in Lublin, Lublin, Poland; ^m^ Clinical Division of Infectious Diseases, University of Warmia and Mazury in Olsztyn, Olsztyn, Poland; ^n^ Department of Infectious Diseases, Regional Hospital, Opole, Poland; ^o^ Department for Adults Infectious Diseases, Medical University of Warsaw, Warsaw, Poland

**Keywords:** antiretroviral treatment, virologic suppression, cART efficacy, WHO target, viral replication, virologic control

## Abstract

**Introduction**: Modern combined antiretroviral therapies (cART) allow to effectively suppress HIV-1 viral load, with the 90% virologic success rate, meeting the WHO target in most clinical settings. The aim of this study was to analyse antiretroviral treatment efficacy in Poland and to identify variables associated with virologic suppression.

**Methods**: Cross-sectional data on 5152 (56.92% of the countrywide treated at the time-point of analysis) patients on cART for more than six months with at least one HIV-RNA measurement in 2016 were collected from 14 Polish centres. Patients’ characteristics and treatment type-based outcomes were analysed for the virologic suppression thresholds of <50 and <200 HIV-RNA copies/ml. CART was categorized into two nucleos(t)ide (2NRTI) plus non-nucleoside reverse transcriptase (NNRTI) inhibitors, 2NRTI plus protease (PI) inhibitor, 2NRTI plus integrase (InI) inhibitor, nucleos(t)ide sparing PI/r+InI and three drug class regimens. For statistics Chi-square and U-Mann Whitney tests and adjusted multivariate logistic regression models were used.

**Results**: Virologic suppression rates of <50 copies/mL were observed in 4672 (90.68%) and <200 copies/mL in 4934 (95.77%) individuals. In univariate analyses, for the suppression threshold <50 copies/mL higher efficacy was noted for 2NRTI+NNRTI-based combinations (94.73%) compared to 2NRTI+PI (89.93%), 2NRTI+InI (90.61%), nucleos(t)ide sparing PI/r+InI (82.02%) and three drug class regimens (74.49%) (*p* < 0.0001), with less pronounced but significant differences for the threshold of 200 copies/mL [2NRTI+NNRTI-97.61%, 2NRTI+PI-95.27%, 2NRTI+InI-96.61%, PI/r+InI- 95.51% and 86.22% for three drug class cART) (*p* < 0.0001). However, in multivariate model, virologic efficacy for viral load <50 copies/mL was similar across treatment groups with significant influence by history of AIDS [OR:1.48 (95%CI:1.01–2.17) if AIDS diagnosed, *p* = 0.046], viral load < 5 log copies/mL at care entry [OR:1.47 (95%CI:1.08–2.01), *p* = 0.016], baseline lymphocyte CD4 count ≥200 cells/µL [OR:1.72 (95%CI:1.04–2.78), *p* = 0.034] and negative HCV serology [OR:1.97 (95%CI:1.29–2.94), *p* = 0.002]. For viral load threshold <200 copies/mL higher likelihood of virologic success was only associated with baseline lymphocyte CD4 count ≥200 cells/µL [OR:2.08 (95%CI:1.01–4.35), *p* = 0.049] and negative HCV status [OR:2.84 (95%CI:1.52–5.26), *p* = 0.001].

**Conclusions**: Proportion of virologically suppressed patients is in line with WHO treatment target confirming successful application of antiretroviral treatment strategy in Poland. Virological suppression rates depend on baseline patient characteristics, which should guide individualized antiretroviral tre0atment decisions.

## Introduction

Antiretroviral treatment leading to virologic suppression is considered not only the most effective treatment option to preserve immune system function but also to decrease the risk of HIV- and non-HIV-associated co-morbidities and death [1–3]. Controlling the HIV replication reduces the risk of HIV transmission and halts the spread of epidemics, as well as limits evolution of the drug resistance, preserving therapeutic options [1,4–7]. The initiation of antiretroviral treatment in all patients with HIV infection, regardless of lymphocyte CD4 count, is recommended by all major treatment guidelines worldwide [8–10].

In Poland, approximately 0.1% of population is infected with HIV with >21,000 diagnosed cases as of January 2017. Approximately ten years ago, the dominant route of transmission shifted from intravenous drug use, with high number HCV coinfections, to men who have sex with men (MSM) with continuous expansion of epidemics in this group [11–13]. Despite the decreasing number of persons who injects drugs intravenously, the increasing number of people combining sex with illicit party drugs – chemsex (especially among MSM from large cities, similar to the previously reported in other European cities [14]), may fuel the HIV epidemics. Antiretroviral medications have been available since 1996, with Polish national HIV/AIDS treatment and prevention programme stably funded since this year. It is currently providing unrestricted and free coverage of all EU antiretroviral registered medications and combinations, as well as genotypic drug resistance, HIV viral load and lymphocyte CD4 assays. Medical and psychological support of HIV-infected people is provided by infectious diseases specialists and specialist teams and is based on the annually updated national treatment guidelines [15].

Modern combined antiretroviral therapies (cART) allow to effectively suppress HIV-1 viral replication in majority of treated cases, with the WHO target of 90% viral suppression among people on antiretroviral therapy to be reached by 2020 [16]. To meet the target, concerted implementation of clinical care with optimized antiretroviral combinations and viral load monitoring is necessary. The aim of this study was to analyse the current real-life treatment efficacy in Poland and to identify variables associated with virologic success.

## Materials and methods

### Study group and inclusion criteria

Cross-sectional data on the antiretroviral treatment efficacy were collected for 5152 patients [(56.92% of total countrywide treated cases (9052 patients) as for 30 June 2016 (data on the number of cases on cART provided by Polish National AIDS Centre, extracted from the national treatment database on this date, and available on request at aids@aids.gov.pl)] followed up in 14/17 Polish HIV treatment centres. The following treatment centres participated in the study (alphabetical order): Białystok (*n* = 259, 5.03% of the study sample), Bydgoszcz (*n* = 344, 6.68%), Chorzów (*n* = 808, 15.68%), Gdańsk (*n* = 476, 9.24%), Kraków (*n* = 399, 7.74%), Lublin (*n* = 74, 1.44%), Łódź (*n* = 320, 6.21%), Opole (*n* = 24, 0.46%), Ostróda (*n* = 41, 0.79%), Szczecin (*n* = 377, 7.32%), Wrocław (*n* = 303, 5.88%), Warsaw [two centres: Hospital for Infectious Diseases (*n* = 1522, 29.54%) and Medical University (*n* = 124, 2.41%)], Zielona Gora (*n* = 81, 1.57%).

Study protocol was approved by the institutional review board named Bioethical Committee of Pomeranian Medical University in Szczecin, Poland (approval number KB-0012/08/12). Research was conducted in accordance with the Declaration of Helsinki. Data for all patients were anonymized. As the patient data were coded and anonymous, and there were no additional procedures associated with this study, no separate written consent was obtained but physicians informed subjects on the planned research and checked for verbal non-opposition from their patients.

Study included participants on stable (uninterrupted) cART, treated for at least six months, with at least one HIV-RNA viral load measurement in 2016. Furthermore, virologic measurement must have been performed after at least six months of uninterrupted antiretroviral therapy. The virological success was defined as HIV-1 viral load either <50 RNA copies/mL or <200 RNA copies/mL with outcomes analysed for these two HIV-RNA thresholds, based on the measurement taken in 2016. The following data were collected: age at HIV diagnosis, gender, date of HIV diagnosis, route of transmission, history of hepatitis C co-infection based on anti-HCV serology (anti-HCV positive/negative, regardless HCV-RNA or HCV treatment status – these data were not collected), history of AIDS (documented in the medical records), baseline HIV viral load (at the care entry), as well as baseline, nadir and the latest lymphocyte CD4 counts. Date of diagnosis was assumed as the date of positive screening HIV test if later confirmed by Western-blot, immunoblotting or positive serum HIV-RNA. As seroconversion time point was often unavailable in the source documentation, therefore data for acute HIV infections were not collected – date of infection was based on the date of the first positive, confirmed HIV test. Baseline lymphocyte CD4 counts and HIV-RNA are defined as the first documented result after diagnosis of HIV. The latest lymphocyte CD4 count was taken as the last recorded value in the medical records. Transmission route was self-defined by the patient. For final analysis the haemophiliac and vertically infected patients were excluded from the route analyses due to the small sample sizes (15 cases of vertical transmission and four with haemophilia).

Collected cART data included drug classes for the current (last) treatment and their combinations [nucleos(t)ide (NRTI) and non-nucleoside reverse transcriptase (NNRTI), protease (PI), integrase (InI), CCR5 and fusion inhibitors]. Data on the antiretroviral treatment history were not collected. For the final analyses the following treatment groups were used: 2NRTI+NNRTI, 2NRTI+PI, 2NRTI+InI, nucleos(t)ide sparing PI/r+InI as well as combined category for all patients treated with three drug class regimens. All data were extracted from the patient files.

### Statistical analyses

Statistical comparisons were performed using the Chi-square tests for categorical variables. As all continuous variables were distributed in the non-linear manner U-Mann Whitney test was used for analyses. Computations were performed with Statistica 12.0 PL software (Statasoft, Poland). HIV viral load at baseline, lymphocyte CD4 count and age were analysed both as continuous variables and as predefined categories. HIV-1 viral load was categorized using a threshold of 5 log copies/mL, baseline and nadir lymphocyte CD4 count with a threshold of 200 cells/µL and last lymphocyte CD4 count of 500 cells/µL. Additionally, age at diagnosis was subdivided into six categories (≤20 years, 21–30, 31–40, 41–50, 51–60 and >60 years of age) similarly to the classification of age categories for European cohorts [17]. To verify variables associated with virologic success multivariate logistic regression model was adjusted for AIDS history, lymphocyte CD4 baseline and nadir <200 cells/µL, last lymphocyte CD4 count <500 cells/µL, HIV viral load at baseline <5 log copies/mL and HIV transmission route.

## Results

### Current antiretroviral treatment combinations

**Figure 1.**
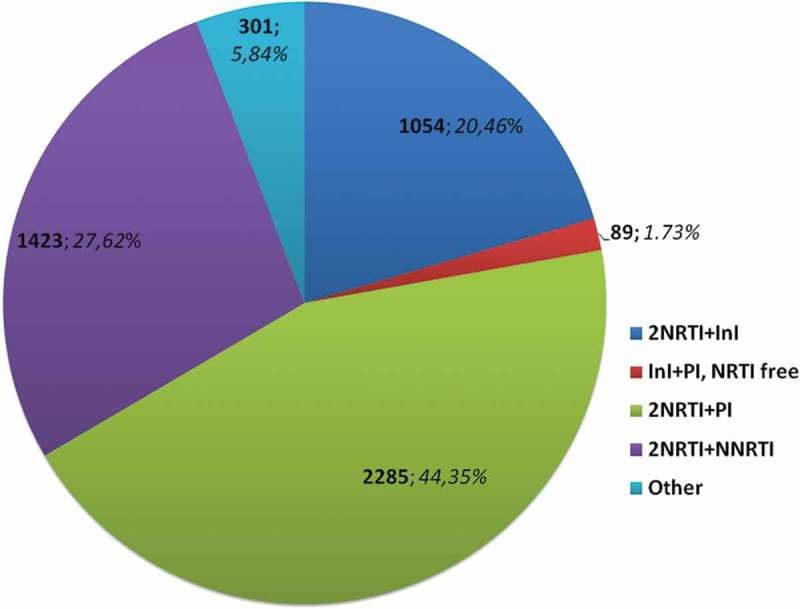
Main antiretroviral regimens used at the time of the last viral load measurement in the analysed data set.

In the analysed data set the most commonly used regimen combined 2NRTI and one PI (*n* = 2285, 44.35%) cases, followed by 2NRTI plus NNRTI (*n* = 1423, 27.62%) and 2NRTI plus integrase inhibitor (InI) (*n* = 1054, 20.46%). NRTI sparing regimen of PI/r+InI was used in 89 (1.73%) patients, while other combinations in 301 (5.84%) individuals (Figure 1). Of the other combinations the most common were three drug combinations of NRTI+PI/r+InI (*n* = 80, 1.55%), NRTI+PI/r+NRTI (*n* = 72, 1.40%) and protease inhibitor monotherapy (*n* = 35, 0.68%) (supplemental Figure 1). As at the time of data collection 196 (3.8%) patients received three drug regimens a separate cumulative category was created for them and added to the statistical analyses.

### Antiretroviral treatment efficacy

Undetectable viral load (<50 copies/mL) was observed in 4672 (90.68%) individuals. Furthermore, among 262 (5.09%) antiretroviral treated patients last HIV-1 viral load was in the range of 50–200 copies/mL indicating 95.77% overall treatment efficacy if threshold <200 copies/mL was adopted (Table 1). At the time of treatment efficacy analysis the majority of individuals presented with lymphocyte CD4 count >500 cells/µL (*n* = 3103, 60.23%). Last lymphocyte CD4 count in the range of 200–499 copies was noted in 1778 (34.51%) cases, and <200 cells/µL among 271 (5.26%) patients. Notably, antiretroviral treatment efficacy was significantly higher, for both HIV-RNA thresholds (<50 copies/mL and <200 copies/mL) for MSM, patients with no history of AIDS, anti-HCV negative, cases with higher lymphocyte CD4 counts at care entry, nadir and last analysis as well as for individuals with lower baseline viral load. Additionally, for the threshold <200 copies/mL male gender was associated with higher virologic success rate (presented in detail in the Table 1).

**Table 1.** Virologic outcomes of the antiretroviral treatment in the entire cohort using the HIV-1 viral load thresholds of 50 copies/mL and 200 copies/mL associated with clinical, epidemiological and virologic variables.

	Last viral load <50 copies/mL	Last viral load > 50 copies/mL	p-value	Last viral load <200 copies/mL	Last viral load > 200 copies/mL	p-value	Total
***Gender, n (%)***							
*Female*	872 (89.25)	105 (10.75)	0.087	913 (93.45)	64 (6.55)	<0.0001	977 (18.96)
*Male*	3800 (91.02)	375 (8.98)		4021 (96.31)	154 (3.69)		4175 (81.04)
***History of AIDS-defining condition (%)***							
*Yes (AIDS)*	1083 (86.23)	173 (13.77)	<0.0001	1175 (93.55)	81 (6.45)	<0.0001	1256 (25.37)
*No (non-AIDS)*	3413 (92.4)	281 (7.6)		3566 (96.51)	129 (3.49		3695 (74.63)
***Dominant transmission route, n (%)^a^***							
*IDU*	1085 (86.52)	169 (13.48)	<0.0001 ^a^	1166 (92.98)	88 (7.02)	<0.0001^a^	1254 (26.11)
*MSM*	2277 (93.24)	165 (6.76)		2383 (97.58)	59 (2.42)		2242 (50.84)
*HET*	985 (90.53)	103 (9.47)		1034 (95.04)	54 (4.96)		1088 (22.65)
*VER*	13 (86.67)	2 (13.33)		14 (93.33)	1 (6.67)		15 (0.31)
*HEM*	4 (100)	0		4 (100)	0		4 (0.08)
***Age at diagnosis, median years (IQR)***	32 (26–39)	31 (25–38)	0.45	32 (26–39)	28 (23–36)	0.007	32 (26–39)
***Age at treatment initiation, median years (IQR)***	35 (29–40)	34 (28–40)	0.73	34 (29–41)	33 (26–38)	0.068	35 (28–40)
***Years on treatment, mean (SD)***	5.8 (4.54)	6.32 (4.83)	0.1	5.83 (4.55)	6.64 (4.81)	0.026	5.87 (4.57)
***HCV coinfection status at data collection, n* (%)**							
*Anti-HCV positive*	1318 (86.65)	203 (13.35)	<0.0001	1418 (93.23)	103 (6.77)	<0.0001	1521 (39.43)
*Anti-HCV negative*	2158 (92.38)	178 (7.62)		2271 (97.22)	65 (2.78)		2336 (60.57)
***Last lymphocyte CD4 cell counts, median (IQR) cells/µl***	558 (398–740)	517 (299–692)	<0.0001	584 (395–742)	455 (258–629)	<0.0001	552 (389–735)
***Last lymphocyte CD4 cell counts <500 cells/µL***							
*Yes*	1819 (88.78)	230 (11.22)	0.0001	1930 (94.19)	119 (5.81)	<0.0001	2049 (39.77)
*No*	2853 (91.94)	250 (8.06)		3004 (96.81)	99 (3.19)		3103 (60.23)
***Lymphocyte CD4 cell counts at baseline, median (IQR) cells/µL***	321 (162–487)	243 (87–459)	<0.0001	318 (157–485)	278 (116–459)	0.04	317 (155–485)
***Baseline lymphocyte CD4 cell counts <200 cells/µL, n (%)***							
*Yes*	1229 (87.79)	171 (12.21)	<0.0001	1333 (95.21)	67 (4.79)	0.036	1400 (31.48)
*No*	2817 (92.45)	230 (7.55)		2941 (96.52)	106 (3.48)		3047 (68.52)
***Nadir lymphocyte CD4 cell counts, median (IQR) cells/µL***	239 (117–350)	204 (67–296)	<0.0001	234 (111–349)	160 (68–296)	<0.0001	248 (109–347)
***Nadir lymphocyte CD4 cell counts <200 cells/µl, n (%)***							
*Yes*	1682 (87.64)	237 (12.36)	<0.0001	1817 (94.73)	101 (5.27)	0.0001	1918 (43.02)
*No*	2368 (93.23)	172 (6.77)		2464 (97.01)	76 (2.99)		2540 (56.98)
***HIV viral load at baseline, median (IQR) log copies/mL***	4.72 (4.21–5.25)	5.02 (4.46–5.48)	<0.0001	4.74 (4.21–5.27)	4.9 (4.36–5.42)	0.05	4.78 (4.2–5.28)
***HIV viral load at baseline > 5 log copies/mL, n (%)***							
*Yes*	1288 (87.62)	182 (12.38)	<0.0001	1402 (95.37)	68 (4.63)	0.079	1470 (38.5)
*No*	2184 (93.02)	164 (6.98)		2266 (96.51)	82 (3.49)		2348 (61.5)

History of AIDS at data collection was available for 4951 cases. Baseline, nadir and current lymphocyte CD4 count for 4458, 4447and 4981 cases, respectively, HCV serology at diagnosis for 3857 patients, HIV-1 viral load for 3818 cases, transmission route for 4803 patients. Age data available for 3023 cases. Current lymphocyte CD4 count and gender available for all (5152) cases.

***^a^****Due to small number of cases in the vertical transmission and haemophiliac groups statistics for the route of transmission calculated for IDU, MSM and HET only.*

IDU, intravenous drug use; MSM, men having sex with men; HET, heterosexual; VER, vertical; HEM, haemophiliac, IQR, Interquartile range.

### Treatment efficacy by age

For 3023 patients with available data on the age at diagnosis, 6.75% (*n* = 204) were ≤20 years of age. For the majority of cases HIV diagnosis was established between 21 and 30 (*n* = 1170, 38.7%) and 31–40 (*n* = 1030, 34.07%) years of age. Diagnoses at ages between 41–50 years were noted among 414 cases (13.7%), while between 51–60 among 155 (5.13%) patients and >60 years among 50 (1.65%) of individuals.

Treatment success rates for the threshold of 50 HIV-RNA copies/mL were similar across all age categories (≤20 years at diagnosis: 87.75%, 21–30 years: 89.74%, 31–40 years: 91.07%, 41–50 years: 88.65%, 51–60 years: 90.97% and >60 years: 88.0%, *p* = 0.28). Applying the <200 copies/mL virologic efficacy threshold, higher rate of treatment failure was observed for patients aged ≤20 years at diagnosis (93.14% with viremia <200 copies/mL) compared to other age categories (21–30 years: 95.47%, 31–40 years: 97.09%, 41–50 years: 96.38%, 51–60 years: 96.13% and >60 years: 100 %, *p* = 0.023). For <200 copies/mL threshold, median age at diagnosis was also notably lower [median: 28 (IQR:23–36)] among virologically failing patients compared to the suppressed ones [median: 32 (IQR: 26–39)], *p* = 0.007.

### Treatment efficacy by the antiretroviral regimen type

**Figure 2.**
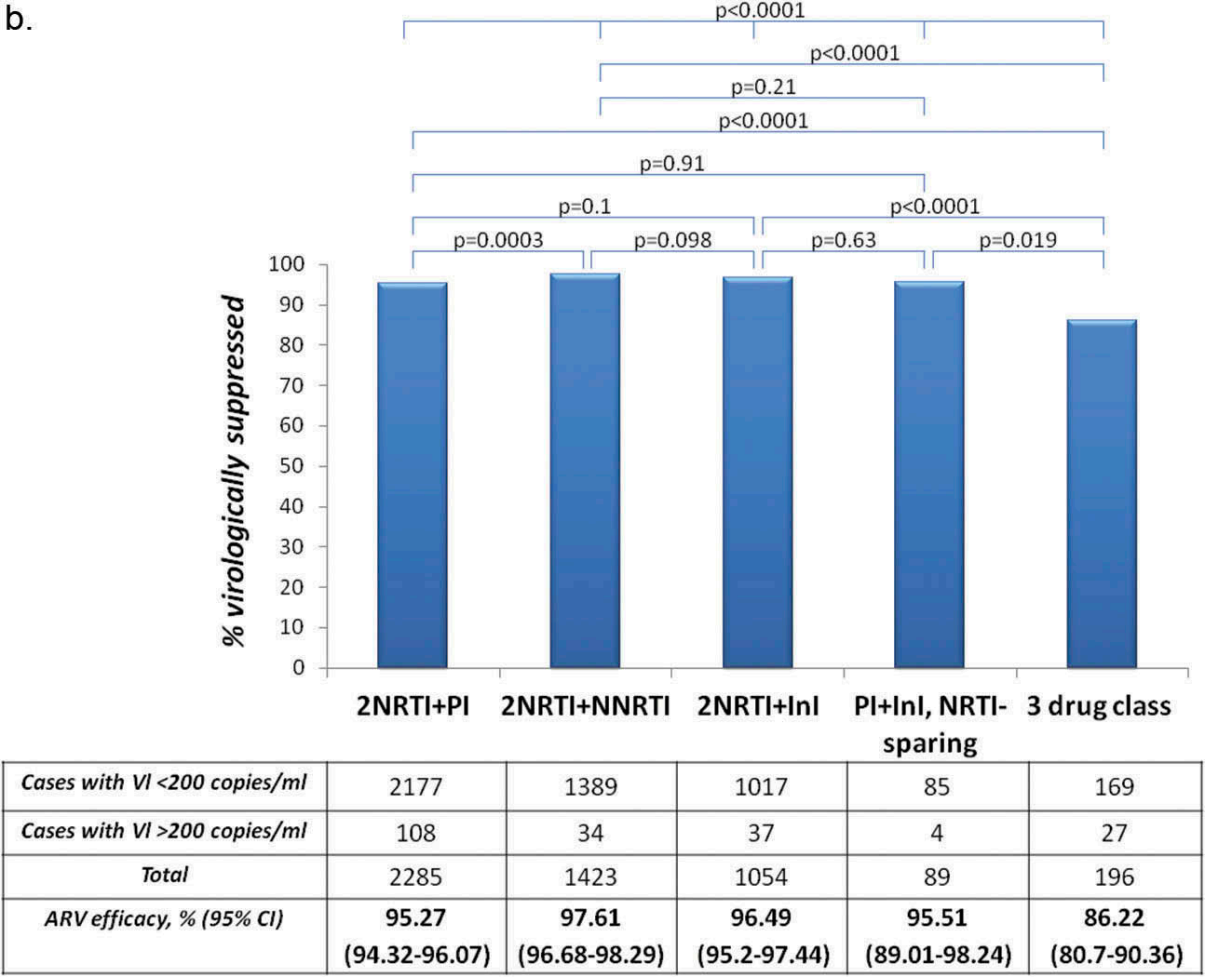
(a) **Virologic success rates (<50 copies/mL) by the last ARV combination. (b) Virologic success rates (<200 copies/mL) by the last ARV combination. For statistics Chi-square test was used.**

For the <50 copies/mL threshold notable differences across regimen types were observed. For this threshold significantly higher virologic success rate was noted for 2NRTI+NNRTI-based combinations (94.73%) compared to 2NRTI+PI (89.93%), 2NRTI+InI (90.61%), nucleos(t)ide sparing PI/r+InI (82.02%) and three drug class regimens (74.49%) (Figure 2(a)). However, for <200 copies/mL threshold, treatment efficacies were similar, with the following virologic success rates: 2NRTI+NNRTI – 97.61%, 2NRTI+PI − 95.27%, 2NRTI+InI – 96.61% and 95.51% for nucleos(t)ide sparing PI/r+InI combinations. Suppression rates <200 copies/mL were consistently and significantly lower for the three drug class regimens (86.22%) (Figure 2(b)). Antiretroviral treatment efficacy for the threshold of 50 copies/mL was notably lower for NRTI sparing PI/r+InI combinations compared to combinations containing two NRTI with either PI, InI or NNRTI (*p* < 0.0001). This difference was less pronounced for the threshold of 200 copies/mL (*p* = 0.0036) (not presented in Figure 2).

**Figure:**
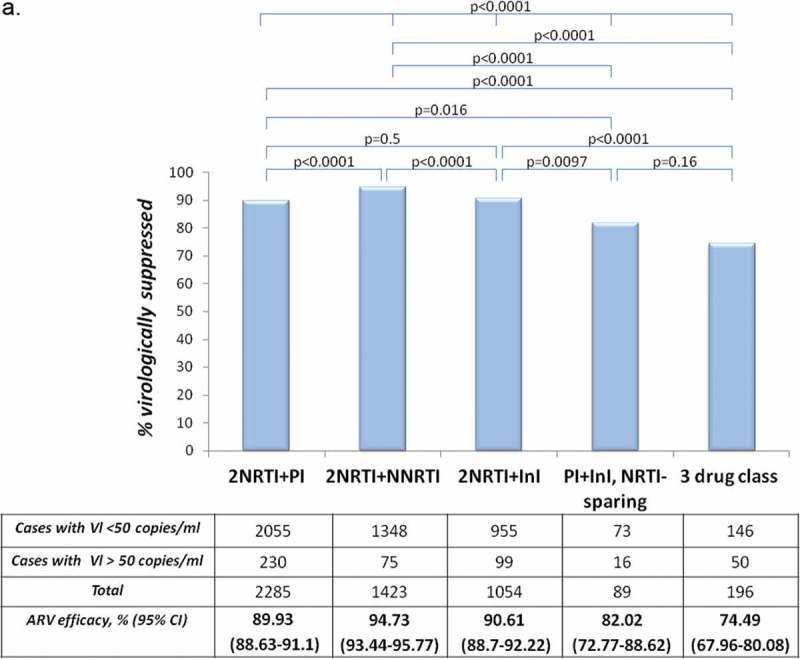

### Treatment efficacy by patient characteristics

Significant differences in the demographic, clinical, immunologic as well as virologic characteristics were observed for the five analysed treatment combinations (2NRTI plus either PI, NNRTI or InI, nucleoside sparing PI/r+InI combinations and three drug regimens) when compared separately (see Table 2 for detailed group size, percentage and statistical data).

**Table 2.** Differences in patient characteristics underlying last treatment option

	2NRTI+PI	2NRTI+NNRTI	2NRTI+InI	PI+InI, nucleos(t)ide sparing	Three drug class treatments	P* 2NRTI+PI vs. 2NRTI+NNRTI	P* 2NRTI+PI vs. 2NRTI+InI	P* 2NRTI+PI vs. PI+InI	P* 2NRTI+PI vs. 3 drug	P* 2NRTI+NNRTI vs. 2NRTI+InI	P* 2NRTI+NNRTI vs. Pi+InI	P* 2NRTI+NNRTI vs. 3 drug	P* 2NRTI+InI vs PI+InI	P* 2NRTI+InI vs 3 drug	P* PI+InI vs 3 drug
***Gender, n (%)***															
*Female*	475 (20.79)	223 (15.67)	174 (16.51)	18 (20.22)	59 (30.1)	0.0001	0.003	0.89	0.002	0.57	0.25	<0.0001	0.36	<0.0001	0.08
*Male*	1810 (79.21)	1200 (84.33)	880 (83.49)	71 (79.78)	137 (69.9)										
***History of AIDS-defining condition (%)***															
*Yes (AIDS)*	648 (29.29)	237 (17.36)	218 (21.82)	25 (29.41)	100 (52.63)	<0.0001	<0.0001	0.98	<0.0001	0.007	0.005	<0.0001	0.1	<0.0001	0.0004
*No (non-AIDS)*	1564 (70.71)	1128 (82.64)	781 (78.18)	60 (70.59)	90 (47.37)										
***Dominant transmission route, n (%)***															
*IDU*	676 (31.63)	252 (18.99)	180 (18.26)	26 (32.5)	85 (48.3)	<0.0001	<0.0001	0.77	<0.0001	0.83	0.013	<0.0001	0.042	<0.0001	0.005
*MSM*	956 (44.74)	784 (59.08)	584 (59.23)	38 (47.5)	47 (26.7)										
*HET*	499 (23.35)	283 (21.33)	219 (22.21)	16 (20.0)	42 (23.86)										
*VER*	2 (0.09)	0	2 (0.2)	0	1 (0.57)										
*HEM*	4 (0.19)	8 (0.6)	1 (0.1)	0	1 (0.57)										
***Age at diagnosis, median years (IQR)***	31 (25–38)	31 (26–38)	32 (25–38)	38 (29–46)	33 (27–40)	0.59	0.33	<0.0001	0.065	0.63	<0.0001	<0.0001	0.0001	0.17	0.014
***Age at treatment initiation, median years (IQR)***	34 (29–40)	28 (28–40)	33 (28–40)	38 (29–46)	36 (30–42)	0.49	0.83	0.001	0.016	0.69	0.0007	0.1	0.002	0.02	0.22
***Years on treatment, median (IQR)***	5 (3–8)	4 (2–8)	2 (1–6)	7 (4–12)	9.5 (6–13)	<0.0001	<0.0001	0.0001	<0.0001	<0.0001	<0.0001	0.008	<0.0001	<0.0001	0.066
***HCV coinfection status at data collection, n* (%)**															
*Anti-HCV positive*	794 (50.8)	311 (27.82)	254 (30.02)	26 (34.67)	92 (56.1)	<0.0001	<0.0001	0.006	0.19	0.28	0.20	<0.0001	0.4	<0.0001	0.002
*Anti-HCV negative*	769 (49.2)	807 (72.18)	592 (69.98)	49 (65.33)	72 (43.9)										
***Last lymphocyte CD4 cell counts <500 cells/µL***															
*Yes*	950 (41.58)	494 (34.72)	427 (40.51)	37 (41.57)	99 (50.51)	<0.0001	0.56	0.99	0.015	0.003	0.18	<0.0001	0.84	0.009	0.16
*No*	1335 (58.42)	929 (65.28)	627 (59.49)	52 (58.43)	97 (49.49)										
***Baseline lymphocyte CD4 cell counts <200 cells/µL, n (%)***															
*Yes*	723 (35.53)	241 (20.55)	281 (31.02)	41 (28.24)	86 (50)	<0.0001	0.017	0.017	0.0002	<0.0001	<0.0001	<0.0001	0.001	<0.0001	0.7
*No*	1312 (64.47)	923 (79.45)	625 (68.98)	44 (51.76)	86 (50)										
***Nadir lymphocyte CD4 cell counts <200 cells/µL, n (%)***															
*Yes*	1043 (50.80)	312 (26.74)	349 (38.69)	50 (58.82)	123 (70.69)	<0.0001	<0.0001	0.14	<0.0001	<0.0001	<0.0001	<0.0001	0.0003	<0.0001	0.06
*No*	1010 (49.20)	855 (73.26)	553 (61.31)	35 (41.18)	51 (29.31)										
***HIV viral load at baseline > 5 log copies/mL, n (%)***															
*Yes*	723 (41.7)	268 (26.56)	323 (40.99)	47 (63.51)	87 (57.62)	<0.0001	0.74	0.0002	0.0002	<0.0001	<0.0001	<0.0001	0.0002	0.0002	0.39
*No*	1011 (58.3)	741 (73.44)	465 (59.01)	27 (36.49)	64 (42.38)										

*p-values calculated for the comparisons between selected regimen combinations.

IDU, intravenous drug use; MSM, men having sex with men; HET, heterosexual; VER, vertical; HEM, haemophiliac, IQR, interquartile range.

Treatment with nucleos(t)ide plus non-nucleoside reverse transcriptase inhibitors was associated with the most favourable clinical, immunological and virologic characteristics compared to other analysed antiretroviral combinations: the least common history of AIDS, the highest baseline, nadir and last lymphocyte CD4 counts, as well as the lowest baseline HIV-1 viral load (supplemental figure 2 a,b,c). Distribution of transmission routes was similar for 2NRTI+NNRTI- and 2NRTI+InI-based treatments.

Among 2NRTI+PI-treated patients AIDS history was notably more common while baseline and nadir lymphocyte CD4 counts were lower compared to 2NRTI+InI, with similar last lymphocyte CD4 count and baseline HIV-1 viral loads. Also the percentage of 2NRTI+PI-treated female as well as anti-HCV-positive individuals was significantly higher compared to 2NRTI+NNRTI and 2NRTI+InI. Age at HIV diagnosis and antiretroviral treatment initiation was similar for all three (2NRTI+PI, 2NRTI+NNRTI, 2NRTI+InI) most common regimens.

Nucleos(t)ide-sparing PI/r+InI combinations were commonly used among patients with history of AIDS as well as injection drug use, both with similar frequency to 2NRTI+PI-based regimens. These patients were notably older at HIV diagnosis compared to any other combination (except for the similar age of the therapy initiation for patients on triple class therapy) and presented with the highest baseline HIV-1 viral loads. Despite the fact that baseline and nadir lymphocyte CD4 count was lower for nucleos(t)ide-sparing PI/r+InI regimens compared to 2NRTI+NNRTI or 2NRTI+InI the last lymphocyte CD4 count was similar to any nucleos(t)ide-based combinations.

It should also be observed that in the group treated with three drug class combinations, the highest percentage of women, individuals with history of AIDS or injection drug use and anti-HCV-positive cases was noted. Also median baseline, nadir and last lymphocyte CD4 count were the lowest in this group compared to any other treatment combination. This group also commonly presented with high viral load, comparable only to the group on nucleoside sparing regimens, with viral load >5 log copies/mL observed in 57.62% of cases and 63.51% for both combinations, respectively.

Lastly, differences for the number of years on antiretroviral treatment were notable across all analysed categories, except between nucleoside sparing PI/r+InI and three drug class regimens. As expected, the shortest time was noted for nucleos(t)ide plus integrase inhibitor [median: 2 (IQR:1–6) years] combinations followed by nucleos(t)ide plus non-nucleoside reverse transcriptase inhibitors [median: 4 (2–8) years], nucleos(t)ide plus protease inhibitors [median: 5 (3–8) years], nucleos(t)ide sparing PI/r+InI [median: 7 (4–12) years] and finally, three drug class treatments [median: 9.5 (6–13) years].

### Variables associated with treatment success in the multivariate model

**Figure 3.**
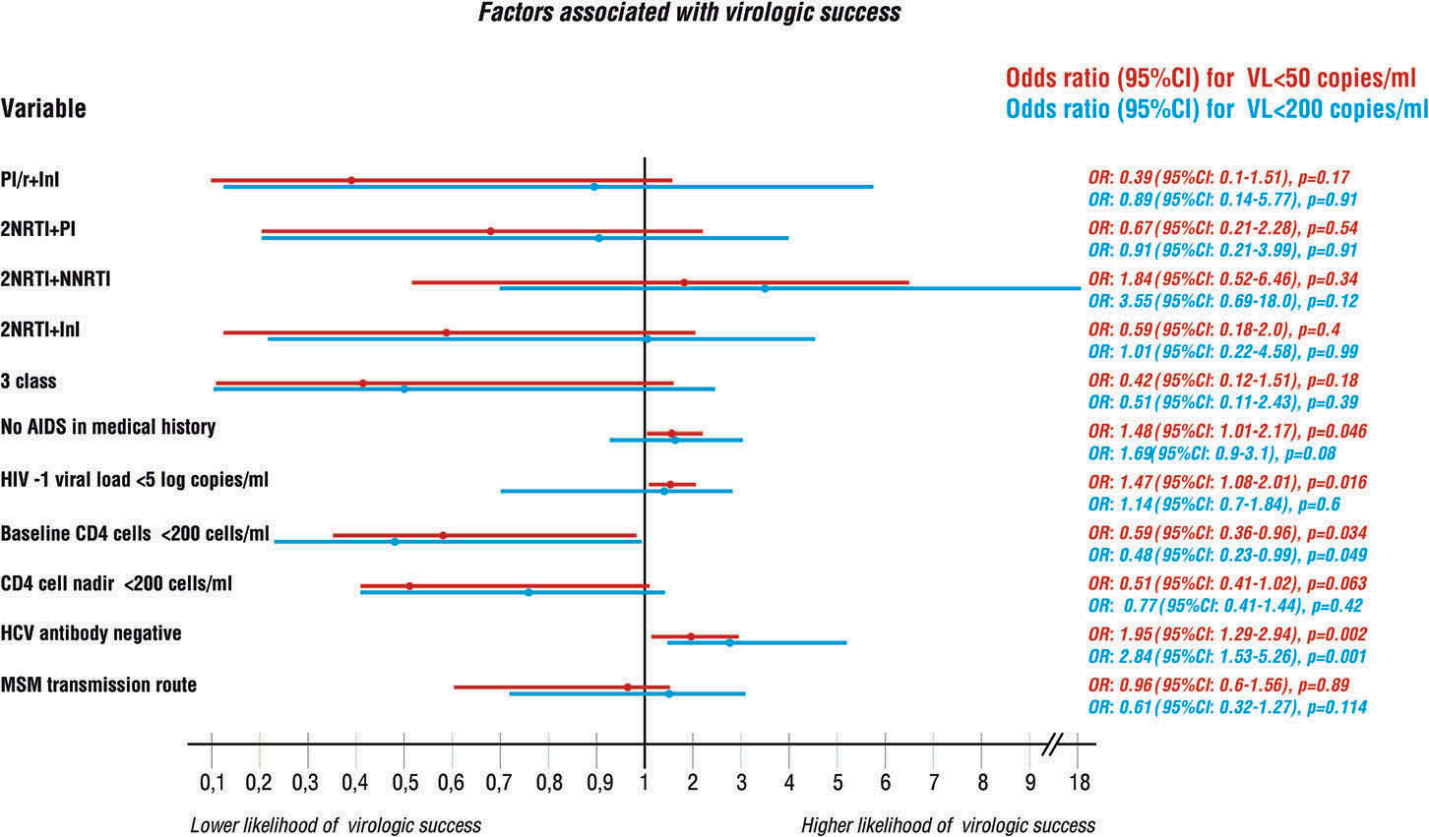
Multivariate logistic regression model presenting factors associated with virologic success for the threshold of 50 (red) and 200 (blue) HIV-RNA copies/mL. Odds ratios, 95% confidence intervals as well as *p*-values are presented on the right.

In multivariate model, adjusted for AIDS history, lymphocyte CD4 baseline and nadir <200 cells/µL, last lymphocyte CD4 < 500 cells/µL, HIV viral load at baseline <5 log copies/mL and transmission route, virologic treatment efficacy, expressed as HIV-1 viral load <50 copies/mL proved similar across the analysed treatment groups (Figure 3). Baseline characteristics significantly influenced the probability of treatment success (defined as HIV-1 viral load <50 copies/mL) with lack of history of AIDS, baseline viral load < 5 log copies/mL, baseline lymphocyte CD4 count ≥200 cells/µL and negative anti-HCV associated with notably better virologic outcomes. For the threshold <200 copies/mL higher likelihood of virologic success was only associated with baseline lymphocyte CD4 count ≥200 cells/µL, and negative anti-HCV serology (Figure 3).

## Discussion

This study presents national data on antiretroviral treatment efficacy for the majority of patients in care in Poland and represents the largest data set published so far, based on 56.92% of all countrywide treated patients for the time of analysis. The highest proportion of cases received nucleos(t)ide backbone treatment combined with PIs, followed by combinations containing NNRTI and InI. These results are similar to other European cohorts [18,19]. Previous published Polish data were limited to single-centre cohort observed until February 2013 and indicated higher frequency (72.4%) of protease inhibitor use with similar percentage of NNRTI used (26.1%) and infrequent treatment with integrase inhibitors (<1.5%) [20]. Integrase inhibitors were introduced in Poland in 2008 with raltegravir, elvitegravir containing regimens in 2012 and dolutegravir in 2014. Our current data reflect the trend for increased use of integrase inhibitors and switching-off from protease inhibitors as indicated by the most recent treatment guidelines [9,15]. Implementation of integrase inhibitors was previously associated with good virologic efficacy, as well as improved treatment safety and tolerability and low risk of transmission of drug resistance [21–23], therefore further increase in the use of this class may be expected in the future.

Antiretroviral treatment efficacy exceeded 90% and 95% for the thresholds of 50 and 200 HIV-RNA copies/mL, respectively. Observed differences in the ratio of virologic success were largely dependent on the baseline patient characteristics and less favourable clinical, immunologic and virologic profile reflected by increased odds ratios of failures associated with the history of AIDS, lower baseline lymphocyte CD4 count, higher HIV viral load at care entry or hepatitis C coinfection and is likely associated with delayed linkage to care. Virologic suppression rates >90% are consistent with the WHO 90–90–90 target and similar to the ones observed for European high income countries such as UK (94% for the threshold of 200 copies/mL) [24], Sweden (94.7% with viral load <50 copies/mL and 98.5% <200 copies/mL) [25], Netherlands (94.2–96.6% for the threshold of 100 copies/mL depending on the number of years on treatment) [26] or ~95% for Switzerland (<200 copies/mL) [27] and were higher compared to some countries such as Georgia (85%) or Japan (87.7%) [28,29]. Presented virologic outcomes indicate high efficacy of the current, free healthcare and antiretroviral treatment access for all Polish citizens and residents. Similar system and outcomes were presented for France, with the virologic success rates of 90.3% (threshold <500 copies/mL, the years 2009–2011) in a large (>80,000 people) data set [19].

In our study virologic efficacy for the threshold of <200 copies/mL was lower for patients aged ≤20 years at diagnosis. Similarly in the Cohere study group, virological success rates were notably lower among individuals aged ≤20 years at diagnosis, which is likely related to adherence issues among adolescents and young adults or may reflect underlying psychological, social, addiction or mental problems in this group [17,30]. In the light of the lifelong antiretroviral treatment this age group requires special clinical focus and treatment optimization to prevent the development of drug resistance and avoid disease progression [31]. Rate of antiretroviral treatment success presented in this study should also be related to the other levels of HIV care cascade in Poland – namely percentage HIV diagnosed and on antiretroviral treatment. Significant gaps exist in this knowledge for the country. Firstly, number of undiagnosed individuals is estimated at ~43% for overall population; however in the recent model, percentage undiagnosed MSM was predicted to reach 69.3% (with 53.9–76.1 confidence interval) [32,33]. Low testing rates were related to existence of barriers to testing among key populations such as MSM and IDUs with large proportion of data on the probable transmission being underreported [34,35]. Also, European Centre for Disease Prevention and Control (ECDC) data estimate that only 63% of people diagnosed received cART as of 2015–2016; however, this number may be underestimated as the number of HIV-infected patients living and receiving treatment in European Union is unknown. Sample presented in the current study may miss the populations with infrequent or irregular follow-up, diagnosed but unlinked to care and most recently diagnosed patients with cART administered for less than six months.

It should be noted that our data revealed notable differences in the frequency of viral suppression rates related to the last antiretroviral treatment option using univariate statistics, mitigated in the multivariate models by the initial clinical (history of AIDS), virologic (HIV-1 viral load at care entry of 5 log copies/mL) and immunologic (baseline lymphocyte CD4 count of 200 cells/µL) patient characteristics and the status of HCV coinfection. Some of the treatment efficacy differences reflect selection of patients with more favourable profile – which is clearly the case of the 2NRTI+NNRTI-based regimens. This group presented with the lowest median HIV-1 viral load, the highest baseline and nadir lymphocyte counts and the least frequent history of AIDS – variables known to affect treatment efficacy [36–38]. Also, as our cross-sectional analysis evaluated only last treatment option, patients with prior virologic failure were likely switched to other combinations, therefore the NNRTI-treated group most likely included stably treated, well-adherent patients. On the other hand, of the most commonly used combinations 2NRTI+PI-treated cases presented with the least favourable clinical profile reflected by high percentage of AIDS diagnoses and low baseline/nadir lymphocyte CD4 counts – comparable to nucleoside-sparing PI/r+InI. Presented data were obtained for the real-life clinical setting with the baseline patient characteristics and data from clinical trials guiding therapeutic decisions may also reflect lower virologic efficacy of boosted protease inhibitor compared to 2NRTI + NNRTI combinations noted in clinical trials [39]. Less favourable outcome related with 2NRTI+InI use compared to 2NRTI+NNRTI may be associated with shorter availability of this drug class and selection of patients with lower lymphocyte CD4 counts and higher HIV-1 viral load at care entry. Most likely, virologic efficacy data from observational cohorts will reflect the favourable outcomes noted in the randomized controlled trials as the use of InI further increases among both antiretroviral treatment naive and experienced patients [40–43].

Due to special clinical interest analysis included two additional treatment groups: nucleoside-sparing PI/r+InI-treated cases and three drug class treated individuals. Switching off the nucleos(t)ide tends to be selected in aging patients with increasing risk of NRTI-related adverse events, especially kidney injury and loss of bone mineral density [44]. Virological outcomes of dual PI/r+InI therapy vary significantly, often with comparable efficacy to the standard nucleos(t)ide-containing triple regimen. However, they were proven to be less efficacious among patients with lymphocyte CD4 nadir <200 copies/mL and HIV-1 viral loads >5 log copies/mL [45–47]. It was also associated with emerging resistance and may limit the subsequent therapeutic options [48,49]. NRTI-sparing PI/r+InI regimens in our study was selected in older patients as reflected by the higher age at HIV diagnosis and antiretroviral treatment initiation in this subgroup, also the length of previous therapy was the longest for these individuals. Notably, this group presented with the highest baseline HIV-1 viral load – a factor mentioned earlier associated with the decreased virologic efficacy for the two-drug NRTI sparing regimens. This might have resulted in the lower frequency of viral load suppression to <50 copies/mL and underscores the necessity for attentive implementation of dual therapy among patients with viremia exceeding 5 log copies/mL.

Finally, combination of at least two active antiretroviral compounds is required to achieve virologic control among treatment-experienced cases. In the setting of treatment failure and emerging drug resistance it may be necessary to combine three or more classes of antiretroviral drugs [19,50–52]. In this analysis we have included the three drug class treated individuals who represent difficult-to-treat cases with likely prior attempts of therapy optimization. This was reflected by the longest time of antiretroviral exposure (median 9.5 years) in this subgroup. The 86.22% suppression rate for the threshold of 200 copies/mL, despite being lower than for other options, seems satisfactory, especially in the light of high frequency of AIDS diagnosis, poor immunologic characteristics, common history of injection drug use and HCV coinfection in this group. It should be emphasized that non-standard regimens presented and discussed earlier were used among more experienced patients as reflected by age and length of follow-up, with possible higher rate of drug resistance adversely influencing treatment success rates [53]. Second-line treatment regimens have been previously associated with decreased virologic efficacy [38,54]. Additionally, some of the observed associations, especially for the NRTI-sparing and >3 drug regimens may be related to the treatment sequencing in highly experienced patients to optimize the treatment for previous resistance or observed toxicity.

The study has the following limitations: Firstly, only the individuals with viral load assessment performed within the last six months from database closure were investigated, which may have led to overestimation of the virological success rates based only on the patients who remained in the close care. Some of early discontinuations might have been missed by this criterion; however, HIV viral loads are assessed every three to six months and such a criterion best reflected clinical practice in Poland. Secondly, for this study it was not possible to assess the history of the antiretroviral regimen changes; therefore, statistics was based on the last recorded antiretroviral treatment combination. This limitation is related to the unavailability of electronic records reflecting treatment changes. Both limitations are at least partially mitigated by the group size which strengthens validity of the presented results.

## Conclusions

To sum up, presented data indicate high efficacy of the antiretroviral treatment in Poland, fully in line with the millennium WHO 90% target and reflects success of comprehensive HIV management in Poland. Differences in the antiretroviral treatment efficacy are based on the patient characteristics and reflect individualized treatment decisions related to variety of clinical conditions such as infection status, age or adherence. It should be noted, however, that presented high frequency of virologic suppression does not result in the decrease of the number of new cases – HIV epidemics in Poland is expanding, especially among MSM [32,35]. While there is a clinical success, further efforts should focus on prevention, testing and linkage to care. Unrestricted and free access to antiretroviral medications allow to maintain high percentage of virologically suppressed individuals.

## Notes

To access the supplementary material to this article please see Supplementary Files under Article Tools online.
